# Changes in the Chemical Composition of Edible Grasshoppers (*Sphenarium purpurascens*) Fed Exclusively with Soy Sprouts or Maize Leaves

**DOI:** 10.3390/insects13060510

**Published:** 2022-05-28

**Authors:** Alicia Reyes-Herrera, Esther Pérez-Carrillo, Genaro Amador-Espejo, Guillermo Valdivia-Nájar, Celeste C. Ibarra-Herrera

**Affiliations:** 1 Tecnologico de Monterrey, Escuela de Ingeniería y Ciencias, Av. Atlixcáyotl 5718, Reserva Territorial Atlixcáyotl, Puebla C.P. 72453, Mexico; a00826573@tec.mx; 2 Tecnologico de Monterrey, Escuela de Ingeniería y Ciencias, Av. Eugenio Garza Sada 2501 Sur, Monterrey N.L. 64849, Mexico; perez.carrillo@tec.mx; 3 CONACYT-IPN Centro de Investigación en Biotecnología Aplicada, Ex-Hacienda San Juan Molino Carretera Estatal Tecuexcomac-Tepetitla Km 1.5, Tlaxcala C.P. 90700, Mexico; ggamadores@conacyt.mx; 4 CONACYT-Unidad de Tecnología Alimentaria, Centro de Investigación y Asistencia en Tecnología y Diseño del Estado de Jalisco, A.C. (CIATEJ), Camino Arenero 1217, El Bajio, Zapopan C.P. 45019, Mexico; gvaldivia@ciatej.mx

**Keywords:** grasshoppers, edible insects, diet modification, amino acids, fatty acids

## Abstract

**Simple Summary:**

One of the most used insects in Mexico is the grasshopper, which is mostly consumed as a snack and collected in open fields where maize or alfalfa is grown. In this study, diet control of grasshoppers has helped to raise some important components such as protein, unsaturated fatty acids, and fiber content. Therefore, diet control could help to obtain insects with a preferred chemical and nutrient composition, making them a more nutritious alternative for human intake. Additionally, this strategy could improve the techno-functional properties of edible insects and their incorporation as ingredients in daily food.

**Abstract:**

In recent times, insects have gained attention because of their nutritional characteristics as well as the environmental advantages of their production. In this research, the effect of the diet of grasshoppers (*Sphenarium purpurascens*) under controlled conditions on their chemical and nutritional content was studied. The insects were divided into two groups: maize leaf-fed grasshoppers (MFG) and soy sprout-fed grasshoppers (SFG). To evaluate the changes in composition, chemical analysis (protein, fiber, fat, ashes, and chitin) was carried out in triplicate according to AOAC procedures, and a Student’s t-test was used to determine any significant differences. The results showed a higher content of crude protein, in vitro protein digestibility percentage, and sum of non-essential amino acids (NEAAs) in the MFG samples compared with the SFG samples. The total dietary fiber, insoluble dietary fiber, soluble dietary fiber, sum of the EAA, non-essential amino acid percentage (EAA%), and biological value percentage (BV%) were higher in the SFG than the MFG, while in the amino acid profile and chitin content, no significant differences were obtained, although an increase in oleic acid in the SFG was observed. In FTIR, a β-sheet appeared in the SFG, which could be related to the low in vitro protein digestibility. The use of a soy sprout diet caused changes in the chemical composition and nutritional content of grasshoppers. This represents an opportunity to improve their nutritional value for commercial interests.

## 1. Introduction

Insects have attracted considerable attention in recent years due to their nutritional value and environmentally friendly advantages. It is estimated that the number of edible species of insects is around 2000 worldwide [1]. Despite the benefits of consuming edible insects, the acceptance of insects as food has been influenced by several factors, such as sensory properties, social environment, personal beliefs, and risks, among others [1,2,3]. In addition, there is an increasing number of articles about edible insects regarding their farming, processing, or nutritional content not only from countries where insects are commonly eaten like African, Asian, and some Latin American countries but importantly from countries where they are seen as exotic foods, such as Germany and the Netherlands [4,5].

One of the most attractive advantages of edible insects is their protein content, which ranges between 40% and 70% of dry matter in some edible insects such as the house cricket (*Gryllodes sigillatus*), *Acheta domesticus*, *Tenebrio molitor*, desert locust (*Schistocerca gregaria*), lesser mealworm (*Alphitobius diaperinus*), African migratory locust (*Locusta migratoria*), and silkworm (*Bombyx mori*), among others [6,7,8,9]. Other authors who evaluated different diets found that the protein content ranged from 36.7% (day 0) to 53.8% (day 4) when different percentages of laying hen feed were substituted with fish meal in *Hermetia illucens* [10,11,12]. The effects of the two diets (alfalfa and maize leaves) on *Sphenarium purpurascens* were evaluated in a previous study, and significant differences in composition were found. The grasshoppers fed with alfalfa increased 10% in their essential amino acid index (EAAI) and biological value (BV) compared with the grasshoppers fed with maize leaves [10,11,12]. Nevertheless, even though this research was the first approach to the effect of diet on nutritional composition on grasshoppers, this study was not performed under controlled conditions.

Likewise, many studies focused on the lipid content in edible insects. The lipid content of edible insects can be influenced by the species, sex, and stage of development [8]. For example, the effect of a diet on the protein and lipid contents was evaluated in the Argentinean cockroach (*Blaptica dubia*), black soldier fly (*H. illucens*), and yellow mealworm (*T. molitor*) [13] The authors tested the following combinations of diets: high protein-high fat content (HPHF), low protein-high fat content (LPHF), high protein-low fat content (HPLF), and low protein-low fat content (LPLF). Meanwhile, a decrease in the protein content in the Argentinean cockroach and black soldier fly (from 69.8% to 37.5%, and 47.8% to 38.3%, respectively) was reported with the LPHF diet, and in the case of the yellow mealworm, the same effect (from 52.4% to 47.5%) was reported with the LPLF diet. On the other hand, an increase in the protein content (from 69.8% to 72.5%, 43.8% to 46.3%, and 47.8% to 53.6%) in the Argentinean cockroach, black soldier fly, and yellow mealworm, respectfully, with the HPHF diet was observed. For the total fatty acids, a maximum increase of 22.8% for the Argentinean cockroach with the LPHF diet was reported. These results show it is necessary to have not only a high-protein diet but also a high-fat diet to modify the protein content in insects [13]. Similarly, other authors evaluated diet modification using lentils and wheat bran on *T. molitor*, including a variation of temperature. The lentil diet administered to *T. molitor* at 15 °C produced the highest increase in crude protein (1.19 times) and more polyunsaturated fatty acids (PUFAs) (1.18 times) than at 25 °C [14,15]. Therefore, there is an impact from the diet on the chemical composition of edible insects, and depending on the composition of the diet, changes will be reflected either in the protein content or the fatty acids content.

One of the problems related to edible insects used as food is their seasonal availability. To tackle this situation, insect farms have been developed to grow different species of insects, such as the silk worm (*B. mori*), vespine wasp (*Vespa magnifica*), flightless cockroach (*Eupolyphaga sinensis*), housefly (*Musca domestica*), black soldier fly (*H. illucens*), mealworm (*T. molitor*), palm weevil (*Rhynchophorus ferrugineus*), tropical banded cricket (*G. sigillatus*), and house cricket (*A. domesticus*), among others [16]. Nonetheless, to produce insects on farms, it is important to consider aspects such as production systems, loss of genetic diversity, safety, stage of development, season, and location [17]. Additionally, different factors such as diet and growth conditions can be studied to conduct the obtention of a preferred product. In this way, the safety and high nutritional quality of edible insects can be assured. Grasshoppers usually consume maize leaves, and soybean sprouts represent an important source of protein (40%) [18]. Therefore, in this work, maize leaves and soybean sprouts were used. The aim of this study is to evaluate the effect of two different diets—soy sprouts or maize leaves (which are common diets)—on the chemical and nutritional composition of grasshoppers (*S. purpurascens*).

## 2. Materials and Methods

### 2.1. Prototype Farm

The diets of the grasshoppers were controlled using a prototype farm, which was built using metal containers with a diameter of 1 m and height of 50 cm. The floor was covered with sawdust, and the container was covered with a plastic net (pores of 1.4 mm). Adult live grasshoppers (*Sphenarium purpurascens*) were purchased in Coronango, Puebla (Mexico) through a local collector from September 2019 to January 2020. They were separated into two groups: the first group was soy sprout-fed grasshoppers (SFG), while the second group was fresh maize leaf-fed grasshoppers (MFG). The soy sprouts were purchased in a local market, and the maize leaves were obtained from a corn field in San Pedro Cholula, Puebla (Mexico). Fresh food was available during the whole experiment period. During this study, the ambient conditions were 20–26 °C and 70.7–82.5% relative humidity, and wet sponges were employed to water the grasshoppers. The grasshoppers of each group were fed for 2 weeks, and then they were drowned in water at room temperature and washed 3 times before being frozen at −80 °C and freeze-dried using a Labconco Freezone 4.5 (Kansas City, MO, USA). After freeze-drying, the grasshoppers were blended using a Nutribullet^®^ NBR-061WM 600W (Los Angeles, CA, USA). This process was repeated eight times. From the grasshopper powder of each group, three samples were taken randomly for further analysis.

The grasshoppers, soy sprouts, and maize leaves were analyzed for their chemical compositions, including protein, crude fat, fiber, ash, and chitin. Additionally, the in vitro protein digestibility, amino acid and fatty acid profiles, and Fourier transform infrared spectrometry analysis of the grasshopper samples were evaluated.

### 2.2. Chemical Composition

The protein content was analyzed by the micro Kjeldahl method according to AOAC method 920.87 using approximately 0.1 g of grasshopper flour. A conversion factor of 5.33 was used to avoid overestimation of the protein in the insects [11,19,20]. For the soy sprouts and maize leaves, 4.64 was used as a conversion factor [21]. The crude fat (AOAC 945.38), dietary fiber (AOAC 996.11 and 2002.02 and AACC 76-31.01) and ashes (AOAC 923.03) were determined according to standardized methods. The chitin was estimated by the method of Black and Schwartz (1950) [22], with some modifications reported by González et al. (2019) [23]. Briefly, 500 mg of grasshopper powder was mixed with 10 mL of 1 M HCl at 85 °C for 50 min under constant stirring. After that, the samples were centrifugated at 3000 *g* for 5 min and then washed with distilled water. The sediment was suspended with 10 mL of 1 M NaOH at 90 °C and mixed for 35 min. Finally, the solids were filtrated using a Buchner funnel with filter paper (pore size: 20–25 µm) and washed with deionized water three times. After drying at 100 °C overnight, the purified insect chitin was calculated by gravimetry.

### 2.3. In Vitro Protein Digestibility

In vitro protein digestibility was evaluated using a multienzymatic technique [24]. Pancreatic porcine trypsin from porcine pancreas type IX-S (T0303, Sigma-Aldrich, St. Louis, MO, USA), α-chymotrypsin from bovine pancreas (C4129, Sigma-Aldrich, St. Louis, MO, USA), and protease from bovine pancreas Type I (P4630, Sigma-Aldrich, St. Louis, MO, USA) were used.

### 2.4. Extraction, Identification, and Quantification of Amino Acids

The grasshopper (*S. purpurascens*) samples were mixed with ethanol and placed in an ultrasonic bath at 30 °C for 60 min. Then, the samples were filtered (Whatman filter no. 1, International Ltd., Maistone, UK), and 0.2 gr of the filtered dried sample was placed into a porcelain vessel, mixed with 5 mL of 6 N HCl, and subjected to microwave-assisted extraction (microwave reaction system Multiwave PRO, Anton Paar GMbH, Graz, Austria) during 24 h at 80 bar, 100 °C, and a constant power of 850 W. Afterward, 1 mL of the sample was diluted with 1 mL of 0.1% formic acid in milli-Q water and preserved in a freezer until analysis. An Acquity I-class UPLC system (Waters, Milford, MA, USA) was used for the analysis, which was equipped with a Waters XBridge^®^ BEH Amide column (150 mm × 3.0 mm, 2.5 µm) (Waters, Dublin, Ireland) and coupled to a Xevo TQ-S micro Tandem Mass Spectrometer (Waters, Milford, MA, USA). The software MassLynx (v4.2; Waters, Manchester, UK) and TargetLynx were used for the control instrument and acquisition of data. Next, 0.5 µL of the sample was injected into the system, and the column temperature was maintained at 35 °C. The mobile phases were 10 mM ammonium formate +0.1% formic acid in acetonitrile (A) and 10 mM ammonium formate +0.1% formic acid in water (B), with a flow rate of 0.4 mL/min. The elution gradient was as follows: 99.9% A as the initial condition, 94.1% A at 6.1 min, 82.4% A at 10.0 min, and 70.6% at 14.0 min. The conditions of the TQ-S micro were as follows: a 1-kV capillary voltage, the ionization source was electrospray in positive mode, the desolvation temperature was 500 °C, the desolvation gas (nitrogen) flow was set at 1000 L/h, and the source temperature was 150 °C. Quantification of the amino acids was carried out using a calibration curve in the range of 10–500 pmol. The essential amino acid index (EAAI) was obtained using a whole egg as a reference, essential amino acids [25], and using the method described in [26]. The Protein Digestibility Corrected Amino Acid Score (*PDCAAS*) was calculated using Equation (1) [27]. The biological value (BV) was calculated according to Equation (2) [28]:

(1) PDCAAS=amino acid score×% true digestibility

(2) BV=1.09×EAAI−11.17

### 2.5. Oil Extraction and Fatty Acid Profile

For oil extraction, 10 g of the grasshopper (*S. purpurascens*) samples were homogenized with 20 mL of *n*-hexane and placed in an ultrasonic bath at 30 °C for 60 min. The samples were filtered (Whatman filter no. 1, International Ltd., Maistone, UK), placed into a 50-mL round-bottom flask, and the residual solvent was removed with a rotary evaporator (IKA RV-10 Digital, Shanghai, China). The extracted oil was transferred to amber flasks using ethanol, and the residual solvent was removed by flushing with nitrogen. Then, the dried film was transmethylated by adding 1 mL of 2 M of KOH in methanol. The samples were vigorously mixed and reposed at room temperature for 5 min. Extraction of the fatty acid methyl esters (FAMEs) was carried out with 1 mL of HPLC-grade *n*-hexane (Sigma Aldrich, St. Louis, MO, USA) twice. Afterward, the hexane was evaporated with a nitrogen stream and reconstituted to 1 mL using HPLC-hexane. The FAME extracts were preserved at −20 °C until analysis.

The FAME content of the grasshopper oil was determined by gas chromatography (GC). An aliquot of 1 μL was injected onto the GC system (G1530N, Agilent Technologies Inc., Santa Clara, CA, USA) equipped with a single quadrupole mass spectrometer detector (MSD 5975) and split and splitless injector (180 °C/2 min), and nitrogen was used as the carrier gas (4 mL/min). The fatty acids were injected into a fused silica capillary column (DB 5% phenyl 95% dimethylpolysiloxane, 30 m × 0.25 mm × 0.25 μm thickness) (Agilent Technologies, Santa Clara, CA, USA) with a temperature program of 180–245 °C at 5 °C/min, followed by holding of 10 min at 245 °C. The total analysis time was approximately 27 min. The FAMEs were relatively quantitated by using external standards (Sigma-Aldrich, Supelco 37 Component FAME Mix-CRM47885, St. Louis, MO, USA). All the samples were prepared separately in duplicate, and analyses were carried out in triplicate.

### 2.6. Fourier Transform Infrared (FTIR) Spectroscopy

The grasshopper samples were analyzed by FTIR. For this determination, an FTIR spectrometer (Vertex 70, Bruker, Rosenheim, Germany) in ATR mode (with a Platinum ATR accessory) in the infrared region between 4000 and 400 cm^−1^ was used. All the data were analyzed using Origin 8.0 (OriginLab, Northampton, MA, USA).

### 2.7. Statistical Analysis

Once all the analyses were performed, a Student’s *t*-test was used to evaluate the significant differences between samples (*p* < 0.05). For this purpose, Minitab^®^ statistical software version 21.1.1 (State College, PA, USA) was used. The average values of the triplicates and standard deviation (SD) are presented in the Results section.

## 3. Results

### 3.1. Chemical Composition

Table 1 shows the composition of the soy sprouts and maize leaves used to feed the grasshoppers. The protein and crude fat contents of the soy sprouts were 1.2% and 2.6% higher than those in the maize leaves, respectfully, and the ash content was 0.6% lower than that in the maize leaves (*p* < 0.05). Meanwhile, the ash content was lower in the soy sprouts than in the maize leaves.

Table 2 shows the compositions of the grasshoppers with two different diets: soy sprouts (SFG) and maize leaves (MFG). Significant differences were found in the chemical compositions, except for the chitin content (*p* < 0.05). While the protein content was higher in the MFG, the rest of the components were obtained in higher proportions in the SFG.

### 3.2. Amino Acid Profile and Protein Quality Indicators

Table 3 shows the amino acid profiles of the grasshopper samples, amino acid scoring patterns (AAS*) for adults [29], and egg amino acid profiles, which are used as a reference [25]. In the case of in vitro protein digestibility, the MFG showed a higher value (77.7%) compared with the SFG (73.4%) (see Table 2). On the contrary, protein quality indicators such as the EAAI, BV, and PDCAAS were higher in the SFG compared with those obtained from the MFG (*p* < 0.05).

### 3.3. Fatty Acid Profile

In Table 4, the fatty acid profiles for the grasshoppers are shown, and in Table 5, the SFAs, monounsaturated fatty acids (MUFAs), PUFAs, and FAMEs are shown. The MUFAs were the predominant fatty acids with 56.8% and 51.2% for the SFG and NFG, respectively. Next were the SFAs with 22.1% and 29.6% for the SFG and NFG, respectively. The fatty acids in lower quantity were PUFAs at 21.0% and 19.1% for the SFG and MFG, respectively. The SFA/UFA ratio was lower for the SFG, and the ω6/ω3 ratio was higher in the SFG than that found in the MFG (*p* < 0.05).

### 3.4. Fourier Transform Infrared (FTIR) Spectroscopy

The α-helix (band position cm^−1^), β-sheet (band position cm^−1^), α-helix and β-sheet turns, and α-helix/β-sheet ratio parameters are presented in Table 6. No significant differences were found in the α-helix/β-sheet ratio of the SFG and MFG (*p* < 0.05).

## 4. Discussion

### 4.1. Chemical Composition

The crude protein content of the SFG was significantly lower than that of the MFG (46.5% and 51.2%, respectively), and subsequently, there could be an effect of the diet on the protein content (see Table 2). These values are lower than those reported previously, which were 65.2% and 75.9% [30,31]. However, in these studies, a conversion factor of 6.25 was used, overestimating the result. Nevertheless, the soy sprouts contained a higher quantity of protein (4.1%, Table 1), and the protein content of the grasshoppers fed with this was lower by 4.7%. This is in accordance with a previous study on the effect of diet using *S. purpurascens*, where 60% and 63.9% of the protein was obtained with alfalfa and maize green fodder diets, respectively (conversion factor of 5.6) [11,32]. In this study, although alfalfa had a higher protein content, the samples that were fed with alfalfa did not show the highest value of protein. These results coincide with other researchers who found that diet is important for the final protein content in insects, and the rise or decrease will depend on the protein content of the diet [14,15,33,34,35]. However, these studies are not conclusive because there is not a defined relation between the protein content in a diet and the protein content in insects. According to the results obtained in this study, a maize-based diet allows protein accumulation in grasshoppers (even when the protein content in the maize leaves is lower than that in soy sprouts; see Table 1). The fat content presented in the SFG was 6.6%, and it was 6.3% for the MFG. These percentages were statistically different. The total dietary fiber (TDF) (Table 2) in the SFG was 32.2%, and it was 24.9% in the MFG, results which are statistically different (*p* < 0.05). This last value was similar to the value reported (23.15%) for grasshoppers fed with maize green fodder [11]. The insoluble dietary fiber (IDF) content was higher in the SFG than in the MFG (29.3% and 23.3%, respectively). The soluble dietary fiber (SDF) content was higher in the SFG (3.8%) than in the MFG (1.5%). However, the soy sprout diet in grasshoppers represents a positive impact since it increased the SDF content, which would allow improving human microbiota health, it would increase the IDFs and the generation of intestinal gas [36].

### 4.2. Amino Acid Profile and Protein Quality Indicators

When observing the amino acid profiles (Table 3), the predominant essential amino acid was leucine, with values of 126.7 and 115.0 mg/g protein in the SFG and MSG, respectively, which were even higher than the 88.2 mg/g protein reported in the egg. This is in accordance with another study, where leucine was the highest among the essential amino acids in *S. purpurascens* (8.1 mg/16 g N) [37]. The contents of nine amino acids were found to be significantly different (*p* < 0.05), with four essential amino acids (EAAs)—leucine, lysine, threonine, and valine—and six non-essential amino acids (NEEA), namely alanine, asparagine, proline, glycine, glutamic acid, and serine. In most of the cases, the SFG presented higher contents of these amino acids than the MFG, except for asparagine and glycine. All AASs were higher than 100%. The results of this study showed that diet modification also had an effect on the profiles of the amino acids and that soy sprout feeding allowed the accumulation of essential amino acids.

The EAAI, biological value (BV), and Protein Digestibility Corrected Amino Acid Score (PDCAAS) (see Table 2) of the SFG (100%, 97.4%, and 22.1, respectively) were higher than those of the MFG (91.3%, 87.8%, and 18.4%, respectively) (*p* < 0.05). These results show that a higher EAAI, BV, and PDCAAS can be obtained using a diet of soy sprouts.

In vitro protein digestibility for the SFG had a value of 73.4%, which was significantly lower than the value for the MFG (77.7%), but it is higher compared with that of the black soldier fly (*H. illucens*), which was between 65.5% and 69.7% [38,39,40]. It has been proven that protein digestibility is related to insoluble fiber because it cannot be degraded nor absorbed in the small intestine [41,42], and this is in accordance with a higher content of insoluble fiber that was found in the SFG (29.3%) than that found in the MFG (23.3%).

### 4.3. Fatty Acid Profile

According to the fatty acid profiles showed in Table 4, the soy sprout diet allowed an increase on important fatty acids for human health such as oleic acid, and this could be confirmed by the ω6:ω3 ratio (see Table 5). (An optimum ratio >20 plays a role in preventing and managing type 2 diabetes, insulin resistance, and neurological disorders [43]) In this study, the ω6/ω3 (59.5/1.71 µg/10 g) ratio was up to 35.1 for the SFG (almost three times higher than in the MFG). The results of the ω6/ω3 ratio in this study were in the range of those in previous studies. According to other studies, the ω6/ω3 ratio went from 1.2 up to 27 in whole insects, and this can be modified through the diet [44]. A ω6/ω3 ratio of 22.8 for male wild *R. differens* and 4.1 with diet changes was reported [35]. The SFA total percentage in the SFG was lower than that of the MFG by up to 7.5% (Table 5), which means that the diet based on soy sprouts decreased the content of SFAs in the grasshoppers. In this study, the predominant SFA in both samples was palmitic acid at 7.7% and 7.9% for the SFG and MFG, respectively (Table 4), making them statistically different (*p* < 0.05). In *R. differens*, the predominant SFA was also palmitic acid (with respect to the total FAMEs), being between 21% and 33%. The MUFAs and PUFAs increased in the SFG in relation to the MFG by 5.6% and 2%, respectively (Table 4). Regarding MUFAs, oleic acid was the most predominant monounsaturated fatty acid in both samples, representing 40.7% and 32.6% for the SFG and MFG, respectively, while α-linoleic acid was the PUFA with the highest content, being 14.5% and 10.3% for the SFG and MFG respectively, showing they were statistically different. Oleic acid and α-linoleic acid are important fatty acids because they are associated with a decreased risk of heart disease and inflammations [35]. In *R. differens*, for MUFAs, the predominant fatty acid was oleic acid (with respect to the total FAMEs) at 15–26%, which coincides with the results in this study, including the percentage of oleic acid doubles in the SFG and MFG. Meanwhile, the SFA/UFA indexes were 0.28 and 0.42 for the SFG and MFG, respectively, and were statistically different (Table 5). For *T. molitor*, the SFA/UFA index was 0.4, but for *H. illucens*, it was 6.56 for both with basal diets (whole wheat meal and wheat bran, respectively). With a modified diet (80% wheat meal or bran and 20% flax seed for *T. molitor* and 80% wheat meal or bran and 20% rapeseed for *H. illucens*), these values diminished to 0.37 and 1.45, respectively. This fact depended on the SFA content in the diet; the higher the SFA content, the higher the SFA/UFA ratio [43]. There are many differences in fatty acid composition, depending on the species, life stage, temperature, light, and diets, but unsaturated fatty acids are more prevalent than saturated fatty acids [33,45]. Feeding insects using different substrates has an impact on their chemical and nutritional content.

### 4.4. Fourier Transform Infrared Spectrometry (FTIR)

According to the results of the α-helix/β-sheet index (Table 6), the predominant structures in both samples were β-sheets, which were less soluble than α-helix, meaning that they formed protein aggregates [46]. This index is correlated to the digestibility of proteins; when there is a higher content of β-sheets, in vitro protein digestibility is lower [47]. In this case, this was confirmed using an in vitro protein digestibility assay that resulted in values of 73.4% and 77.7% for the SFG and MFG, respectively (Table 2), as was explained before. In addition, there were signals between the bands at 1633 and 1637 cm^−1^ for the SFG and MFG, respectively, and these bands are representative of chitin as well as a CH bend and CH_3_ symmetrical deformation, confirming the presence of α-chitin in the samples.

## 5. Conclusions

According to these results, soy sprout-fed grasshoppers could be used as ingredients to improve essential amino acid profiles (EAAI, BV, and PDCAAS) and increase the unsaturated fatty acid content compared with saturated fatty acids or to increase the content of soluble dietary fiber in some foods. The nutritional quality of insects can be enhanced with a proper diet. In future studies, diets including other nutrient sources could be tested to evaluate if the protein content, essential amino acid profile, in vitro protein digestibility, fatty acid profile, and soluble dietary fiber content can be increased using different edible insects. In this way, the use of edible insects as ingredients in different food products can be motivated. Similarly, farms, under the controlled feeding of insects, could be used to assure the nutritional quality and safety of insects to be used as food ingredients.

## Figures and Tables

**Table 1:** Chemical compositions of soy sprouts and maize leaves used to feed grasshoppers.

Component	Soy Sprouts (% *w*/*w*)	Maize Leaves (% *w*/*w*)
Crude protein	4.1 ^a^ ± 0.0	2.9 ^b^ ± 0.0
Crude fat	3.3 ^a^ ± 0.3	0.7 ^b^ ± 0.0
Ashes	0.4 ^a^ ± 0.3	1.0 ^b^ ± 0.1

Data are explained as mean ± SD. Means in each row followed by different letters are significantly different (*p* < 0.05).

**Table 2:** Chemical composition, chitin, in vitro protein digestibility, and protein quality indicators of SFG and MFG.

Component	SFG (% *w*/*w*)	MFG (% *w*/*w*)
*Chemical composition*		
Crude protein	46.5 ^a^ ± 0.8	51.2 ^b^ ± 1.0
Total dietary fiber	32.2 ^a^ ± 1.0	24.9 ^b^ ± 0.4
Insoluble dietary fiber (IDF)	29.3 ^a^ ± 1.4	23.3 ^b^ ± 0.5
Soluble dietary fiber	3.8 ^a^ ± 0.4	1.5 ^b^ ± 0.2
Crude fat	6.6 ^a^ ± 0.1	6.3 ^b^ ± 0.1
Ashes	3.4 ^a^ ± 0.3	2.4 ^b^ ± 0.4
*Chitin analysis*		
Chitin	8.9 ^a^ ± 1.1	9.0 ^a^ ± 1.2
Percentage of chitin of IDF content	30.4 ^a^ ± 0.9	38.6 ^b^ ± 1.2
*In vitro protein digestibility analysis*		
*In vitro* protein digestibility (%)	73.4 ^a^ ± 3.3	77.7 ^b^ ± 1.9
Sum of EAA (mg/g of protein)	492.7	456.6
Sum of NEAA (mg/g of protein)	516.7	617.2
EAAI (%)	100 ^a^	91.3 ^b^
BV (%)	97.4 ^a^	87.8 ^b^
PDCAAS	22.1 ^a^	18.4 ^b^

Data are explained as mean ± SD. Means in each row followed by different letters are significantly different (*p* < 0.05). SFG: soy sprout-fed grasshoppers, MFG: maize leaf-fed grasshoppers; IDF: insoluble dietary fiber; EAA: essential amino acids; NEAA; non-essential amino acids; EAAI: essential amino acid percentage; BV: biological value; PDCAAS: Protein Digestibility Corrected Amino Acid Score.

**Table 3:** Amino acid profile of soy sprout-fed grasshoppers (SFG) and maize leaf-fed grasshoppers (MFG).

	Samples				
Amino Acid	SFG		MFG		Egg
	mg/g of Protein	AAS *	mg/g of Protein	AAS *	mg/g of Crude Protein
*Essential amino acid (EAA)*					
Histidine	24.4 ^a^ ± 1.0	152.3%	21.6 ^a^ ± 0.4	135.2%	24.3
Isoleucine	63.4 ^a^ ± 0.7	211.5%	59.1 ^a^ ± 0.3	197.1%	62.9
Leucine	126.7 ^a^ ± 1.0	207.8%	115.0 ^b^ ± 1.3	188.6%	88.2
Lysine	65.7 ^a^ ± 0.4	136.9%	60.6 ^b^ ± 1.4	126.2%	70.0
Threonine	53.2 ^a^ ± 0.2	212.7%	48.0 ^b^ ± 1.3	192.0%	51.1
Tryptophan	9.1 ^a^ ± 13.2	137.6%	11.0 ^a^ ± 0.0	166.3%	68.5
Valine	92.2 ^a^ ± 0.5	230.5%	83.9 ^b^ ± 1.5	209.7%	58.5
*Non-essential amino acid*					
Alanine	207.0 ^a^ ± 0.5		181.1 ^b^ ± 3.5		59.2
Arginine	71.8 ^a^ ± 0.1		63.8 ^a^ ± 1.5		61.0
Asparagine	7.3 ^a^ ± 0.0		13.3 ^b^ ± 0.0		42.9
Glutamine	0.0 ^a^ ± 0.0		0.0 ^a^ ± 0.0		127.4
Proline	108.3 ^a^ ± 0.2		95.3 ^b^ ± 1.6		42.6
Glycine	6.1 ^a^ ± 13.5		158.8 ^b^ ± 7.3		44.2
Glutamic acid	128.5 ^a^ ± 0.9		118.6 ^b^ ± 0.9		
Serine	116.2 ^a^ ± 3.1		105.0 ^b^ ± 0.3		76.5

Means of repeated analyses (*n* = 3) in columns followed by different letters are significantly different (*p* < 0.05). SFG: soy sprout-fed grasshoppers; MFG: maize leaf-fed grasshoppers; AAS*: amino acid scoring patterns for adults (amended values from the 2007 WHO/FAO/ONU report). Whole hen egg (FAO, 1970) was used as a reference.

**Table 4:** Fatty acid profile for soy sprout-fed grasshoppers (SFG) and maize leaf-fed grasshoppers (MFG).

		Samples	
Fatty Acid		SFG	MFG
		(%)	(%)
Caproic acid	C6:0	0.91 ^a^ ± 0.20	0.75 ^a^ ± 0.08
Caprylic acid	C8:0	1.15 ^a^ ± 0.06	2.40 ^b^ ± 0.36
Capric acid	C10:0	0.66 ^a^ ± 0.02	1.14 ^a^ ± 0.17
Undecanoic acid	C11:0	0.33 ^a^ ± 0.02	0.44 ^b^ ± 0.05
Lauric acid	C12:0	0.72 ^a^ ± 0.02	1.13 ^b^ ± 0.12
Tridecanoic acid	C13:0	0.35 ^a^ ± 0.01	0.79 ^a^ ± 0.17
Myristoleic acid	C14:0	0.38 ^a^ ± 0.01	0.61 ^b^ ± 0.06
Pentadecanoic acid	C15:0	0.47 ^a^ ± 0.00	0.64 ^b^ ± 0.05
Palmitic acid	C16:0	7.71 ^a^ ± 0.12	7.99 ^a^ ± 0.33
Heptadecanoic	C17:0	1.14 ^a^ ± 0.09	1.42 ^b^ ± 0.07
Stearic acid	C18:0	3.44 ^a^ ± 0.10	3.58 ^b^ ± 0.21
Cis-11-eicosenoic acid	C20:0	1.00 ^a^ ± 0.02	2.21 ^b^ ± 0.49
Heneicosanoic acid	C21:0	0.42 ^a^ ± 0.02	0.71 ^b^ ± 0.07
Behenic acid	C22:0	0.88 ^a^ ± 0.09	1.60 ^b^ ± 0.13
Tricosanoic	C23:0	0.49 ^a^ ± 0.07	0.78 ^a^ ± 0.12
Lignoceric acid	C24:0	2.09 ^a^ ± 0.13	3.43 ^b^ ± 0.39
Myristic acid	C14:1	1.19 ^a^ ± 0.00	1.45 ^a^ ± 0.1
Pentadecenoic acid	C15:1	0.33 ^a^ ± 0.00	0.56 ^a^ ± 0.06
Palmitoleic acid	C16:1	4.30 ^a^ ± 0.07	4.31 ^b^ ± 0.09
Heptadecenoic acid	C17:1	0.36 ^a^ ± 0.00	0.60 ^b^ ± 0.07
Oleic acid	C18:1	40.78 ^a^ ± 0.56	32.62 ^b^ ± 2.07
Elaidic acid	C18:1	2.51 ^a^ ± 0.03	2.70 ^a^ ± 0.15
Erucic acid	C24:1	7.02 ^a^ ± 0.40	8.41 ^a^ ± 0.75
Nervonic acid	C24:1	0.36 ^a^ ± 0.00	0.59 ^b^ ± 0.07
γ-linolenic acid	C18:2	0.42 ^a^ ± 0.02	0.83 ^b^ ± 0.07
α-linoleic acid	C18:2	14.54 ^a^ ± 0.29	10.28 ^a^ ± 0.88
Arachidonic acid	C20:4	0.59 ^a^ ± 0.04	0.72 ^a^ ± 0.07
EPA	C20:5	0.37 ^a^ ± 0.01	0.71 ^b^ ± 0.06
α-linolenic acid	C18:3	0.74 ^a^ ± 0.03	0.69 ^a^ ± 0.06
Linolelaidic	C18:2	0.62 ^a^ ± 0.02	0.80 ^a^ ± 0.09
Eicosatrienoic acid	C20:3	2.76 ^a^ ± 0.10	3.88 ^b^ ± 0.37
DHA	C22:6	0.64 ^a^ ± 0.09	0.66 ^a^ ± 0.06
11,14-eicosadienoic acid	C20:2	0.35 ^a^ ± 0.00	0.57 ^b^ ± 0.06

Means of repeated analyses (*n* = 6) in columns followed by different letters are significantly different (*p* < 0.05). SFG: soy sprout-fed grasshoppers; MFG: maize leaf-fed grasshoppers; %: fatty acid percentage in relation to total FAMEs; EPA: eicosapentaenoic acid; DHA: docosahexaenoic acid.

**Table 5:** Fatty acid quality indicators of SFG and MFG.

	Samples	
Parameters	SFG	MFG
SFAs (%)	22.1 ^a^ ± 0.7	29.6 ^b^ ± 3.7
MUFAs (%)	56.8 ^a^ ± 2.5	51.2 ^b^ ± 3.2
PUFAs (%)	20.8 ^a^ ± 0.7	19.1 ^b^ ± 0.7
Total FAMEs (µg/10 g)	341.7 ^a^ ± 109.4	294.2 ^b^ ± 80.8
SFA/UFA ratio	0.28 ^a^ ± 0	0.42 ^b^ ± 0
ω6/ω3 ratio	35.1 ^a^ ± 0.5	13.2 ^b^ ± 0.5

SFG: soy sprout-fed grasshoppers; MFG: maize leaf-fed grasshoppers. Means in each row followed by different letters are significantly different (*p* < 0.05). Saturated fatty acids (SFAs), monounsaturated fatty acids (MUFAs), and polyunsaturated fatty acids (PUFAs) are presented as percentages in relation to total fatty acid methyl esters (FAMEs).

**Table 6:** Assignment of secondary structure to the Gaussian curves obtained from amide I peak through FTIR analysis of SFG and MFG.

Parameter	SFG	MFG
α-helix (band position cm^−1^)	1655	1652
β-sheet band position (cm^−1^)	1617 1631 1679	1621 1636
Turns	1655 1672	-
α-helix/β-sheet	0.88 ^a^ ± 0.009	0.88 ^a^ ± 0.003
R^2^	0.962	0.946

SFG: soy sprout-fed grasshoppers; MFG: maize leaf-fed grasshoppers. Means in each row followed by different letters are significantly different (*p* < 0.05). Data are presented as absorbance intensity ratio (α-helix/β-sheet), where α-helix intensity was taken at 1654 cm^−1^, and β-sheet was taken at 1638 cm^−1^. R^2^ was adjusted for deconvolution.

## Data Availability

The data presented in this study are available within the article.
